# Single cell RNA sequencing reveals distinct clusters of Irf8-expressing pulmonary conventional dendritic cells

**DOI:** 10.3389/fimmu.2023.1127485

**Published:** 2023-05-12

**Authors:** Adan Chari Jirmo, Ruth Grychtol, Svenja Gaedcke, Bin Liu, Stephanie DeStefano, Christine Happle, Olga Halle, Joao T. Monteiro, Anika Habener, Oliver D. Breiholz, David DeLuca, Gesine Hansen

**Affiliations:** ^1^ Department of Pediatric Pneumology, Allergology and Neonatology, Hannover Medical School, Hannover, Germany; ^2^ Biomedical Research in Endstage and Obstructive Lung Disease Biomedical Research in Endstage and Obstructive Lung Disease (BREATH), Member of the German Center for Lung Research (DZL), Hannover, Germany; ^3^ Excellence Cluster Resolving Infection Susceptibility RESIST (EXC 2155), Deutsche Forschungsgemeinschaft, Hannover Medical School, Hannover, Germany; ^4^ Research Core Unit Genomics (RCUG), Hannover Medical School, Hannover, Germany

**Keywords:** conventional dendritic cells, single cell RNA sequencing, inflammation, tolerance, asthma

## Abstract

A single population of interferon-regulatory factor 8 (Irf8)-dependent conventional dendritic cell (cDC type1) is considered to be responsible for both immunogenic and tolerogenic responses depending on the surrounding cytokine *milieu*. Here, we challenge this concept of an omnipotent single Irf8-dependent cDC1 cluster through analysis of pulmonary cDCs at single cell resolution. We report existence of a pulmonary cDC1 cluster lacking Xcr1 with an immunogenic signature that clearly differs from the Xcr1 positive cDC1 cluster. The Irf8^+^Batf3^+^Xcr1^-^ cluster expresses high levels of pro-inflammatory genes associated with antigen presentation, migration and co-stimulation such as *Ccr7*, *Cd74*, *MHC-II*, *Ccl5*, *Il12b* and *Relb* while, the Xcr1^+^ cDC1 cluster expresses genes corresponding to immune tolerance mechanisms like *Clec9a*, *Pbx1*, *Cadm1*, *Btla* and *Clec12a*. In concordance with their pro-inflammatory gene expression profile, the ratio of Xcr1^-^ cDC1s but not Xcr1^+^cDC1 is increased in the lungs of allergen-treated mice compared to the control group, in which both cDC1 clusters are present in comparable ratios. The existence of two distinct Xcr1^+^ and Xcr1^-^ cDC1 clusters is furthermore supported by velocity analysis showing markedly different temporal patterns of Xcr1^-^ and Xcr1^+^cDC1s. In summary, we present evidence for the existence of two different cDC1 clusters with distinct immunogenic profiles *in vivo*. Our findings have important implications for DC-targeting immunomodulatory therapies.

## Introduction

As sentinels of the immune system, dendritic cells (DCs) play a critical role in mounting specific immune responses against pathogens while maintaining tolerance against autoantigens and innocuous environmental antigens like allergens (1, 2). DCs are a heterogeneous cell population with different subtypes that vary regarding phenotype, function and localization (3–6). Based on lineage determining transcriptional programs and functions, conventional DCs (cDCs) are subdivided into two major populations, type 1 (cDC1) and type 2 (cDC2) (7–10).

Type 1 conventional dendritic cells (cDC1) express Xcr1 and their differentiation is controlled by the transcription factors Irf8 and Batf3. On the other hand, cDC2s are characterized by expression of CD11b and SIRPα/CD172α as well as the transcription factor Irf4 (11–13). Although all cDCs are professional antigen processing and presenting cells, cDC1 and cDC2 cells differ in function. cDC2s are crucial for the induction of CD4^+^ T helper cell 2 (Th2) responses (12, 14, 15), while cDCs1 have superior cross-presenting potential and induce cytotoxic T cells against virus-infected cells or tumor cells and are therefore explored as tools for anti-virus or anti-tumor vaccination strategies (16–21). Furthermore, cDC1 are important for tolerance inception by inducing regulatory T cells and suppressing Th2 responses by IL-10 production (22–25).

Many studies have shown the dual tolerogenic or non-tolerogenic ability of cDC1 (17, 19, 24, 26–28), but the overall mechanisms driving cDC1’s dichotomous abilities are still not well defined. This raises the question whether two populations of cDC1 exist or whether functional diversity, i.e. induction of either immunity or tolerance, is completely shaped by the respective immune milieu, as suggested previously (24, 26–30).

In this study, we examine this question by analyzing pulmonary conventional dendritic cells at single cell level to interrogate heterogeneity within cDC1 and cDC2 populations based on well characterized transcriptional patterns (8, 12, 15, 26, 31). We additionally use oligonucleotide barcoded anti-Clec12A antibody to analyze the surface expression of the C-type lectin Clec12A on cDCs since, C-type lectins are involved in enhancing or dampening immune responses (32–37). Clec12A in particular, is associated with tolerance induction due to its triggering-ability of signaling *via* immune-receptor tyrosine-based inhibitory motif (ITIM) (32, 38).

In addition, we use murine models of pro-inflammatory T helper cell 2 (Th2) allergic asthma or anti-inflammatory allergen tolerance to investigate the contribution of distinct cDC subsets in the context of *in vivo* Th2-driven inflammation or tolerance.

Lastly, we apply velocity analysis of single-cell transcriptome data to explore directional trajectories of distinct DC clusters identified by single-cell transcriptome profiles.

Our findings challenge the concept of a single cDC1 population and support the existence of at least phenotypically distinct cDC1 clusters characterized by different expression levels of Xcr1. In our study, the two major Irf8-expressing cDC1 clusters identified by transcriptomic and velocity analysis show a tolerogenic (Xcr1^+^ cDC1s) or pro-inflammatory (Xcr1^-^ cDC1s) gene expression profile and velocity analysis of single-cell RNAseq data support distinct directional trajectories and cell fate.

## Results

### ScRNA sequencing reveals diversity in Irf8-expressing cDC1 both in steady state and under inflammatory conditions

**Figure 1:**
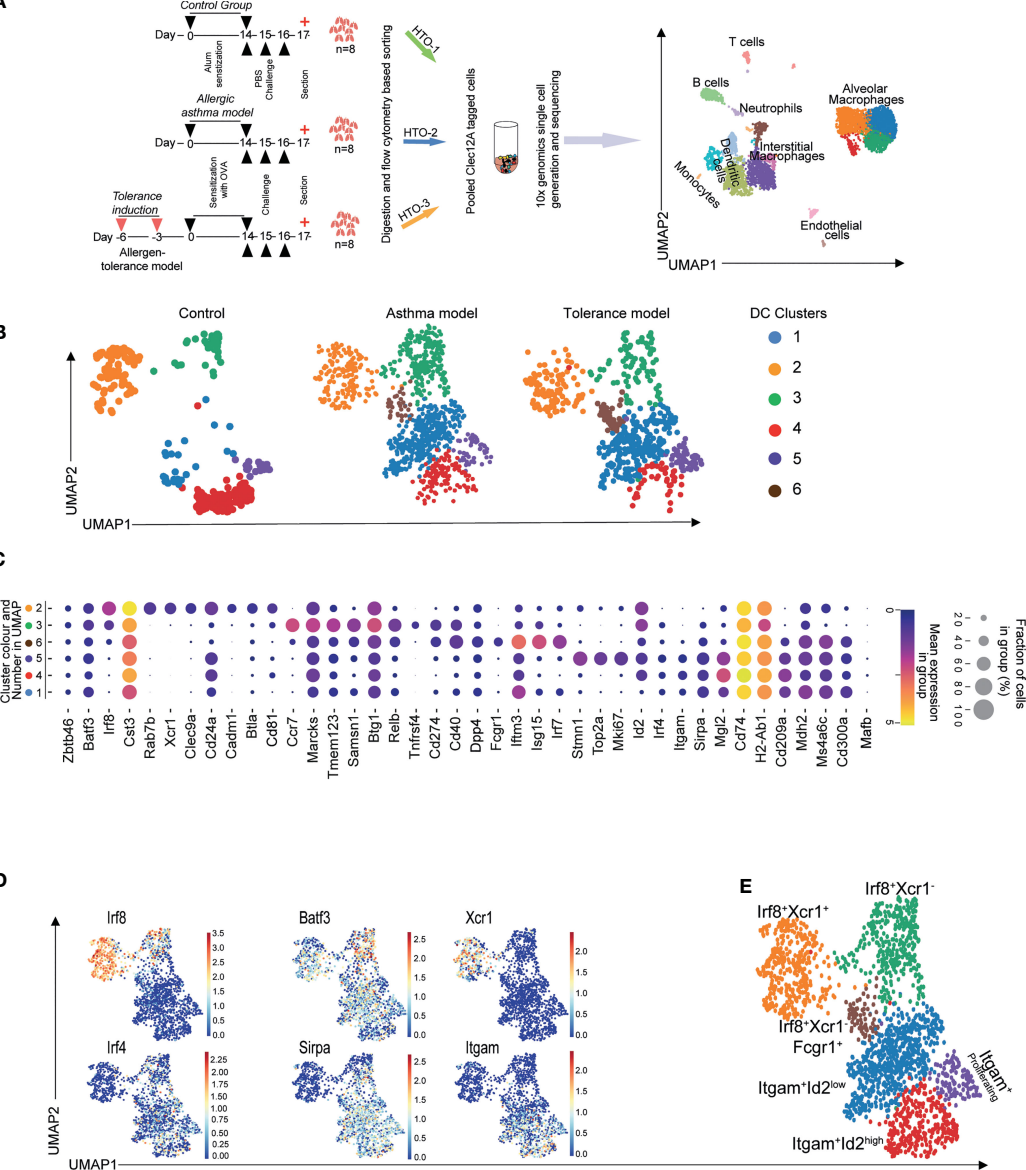
scRNASeq reveals heterogeneity of murine pulmonary cDC. Macrophages and cDCs were sorted from mice subjected to the OVA-induced model of asthma or tolerance as well as allergen-naïve control mice followed by 10x genomics platform based single cell generation and sequencing **(A)**. Unsupervised clustering shows various populations which were identified based on their transcriptomic patterns **(A)**. Clustering revealed multiple DC subpopulations in all treatment conditions **(B)**. cDC subsets determining transcriptomic signatures **(C, D)** were used to annotate Irf8 and Irf4 dependent cDC populations **(E)**. Lungs from n=8 animals were pooled, digested and single cells sorted. Technical replicates were done for the 10X genomics based single cell generation and subsequent sequencing.

We subjected mice to an OVA-induced experimental allergic asthma or allergen tolerance model and sorted conventional dendritic cells (cDCs) and macrophages from the lungs (**Supplementary Figure S1A**) of these as well as allergen-naive control animals for single cell RNA sequencing using the 10X Genomics platform (**Figure 1A**). As shown in **Figure 1A**, unsupervised clustering and dimension reduction presentation using Uniform Manifold Approximation and Projection (UMAP) identified multiple cell populations which we could annotate based on various lineage specific transcriptomic patterns shown in **Supplementary Figure S1**. Further clustering of DCs resulted in six sub-clusters in the lungs of the mice subjected to the experimental allergen or tolerance model whereas there were only five sub-clusters in the lungs of control animals (**Figure 1B**). Clusters 2 and 3 were identified as cDC1 due to their expression of *Batf3* and *Irf8* while, clusters 1,4 and 5 showed *Irf4*, *Itgam* and *Sirpa* expression and thus classified as cDC2s (**Figure 1C**). Whereas all cDC2 clusters expressed *Itgam* and *Sirpα* as expected (**Figure 1D**), surprisingly, *Xcr1* which is characteristically expressed on the cDC1 subset was not expressed by all the clusters expressing Irf8^+^ and Batf3^+^ (**Figures 1D, E**). Also, in the lungs of animals treated with allergen in order to induce either experimental asthma or tolerance, we found an additional cDC population (Cluster 6, **Figure 1B**) which expressed *Irf8*, *Batf3* as well as low levels of *Irf4*, *Sirpa* and *Itgam* (**Figure 1C**). Furthermore, alongside this ambiguity for subpopulation defining genes, this cluster was exclusively positive for *Fcgr1* and showed high expression of genes associated with interferon-response (**Figure 1C**) and thereby resemble a previously described population of inflammatory cDC2 (30, 39). We therefore annotated cluster 6 as Xcr1^-^Irf8^+^Fcgr1^+^ cDC2 (**Figure 1E**). In summary, our data confirmed previously described heterogeneity of cDC2 populations under conditions of experimental allergic airway disease, allergen tolerance and control conditions (40). Moreover, using single-cell resolution, we also demonstrate existence of two clusters of cDC1 in steady state which can be differentiated based on expression of Xcr1. In addition, we identified and confirmed a recently published inflammatory cDC2 population with a high expression of *Fcgr1* and interferon-inducing genes in the lungs of the allergen-exposed animals (39).

### Expression of Xcr1 defines a cDC1 cluster with a tolerogenic transcriptome signature

**Figure 2:**
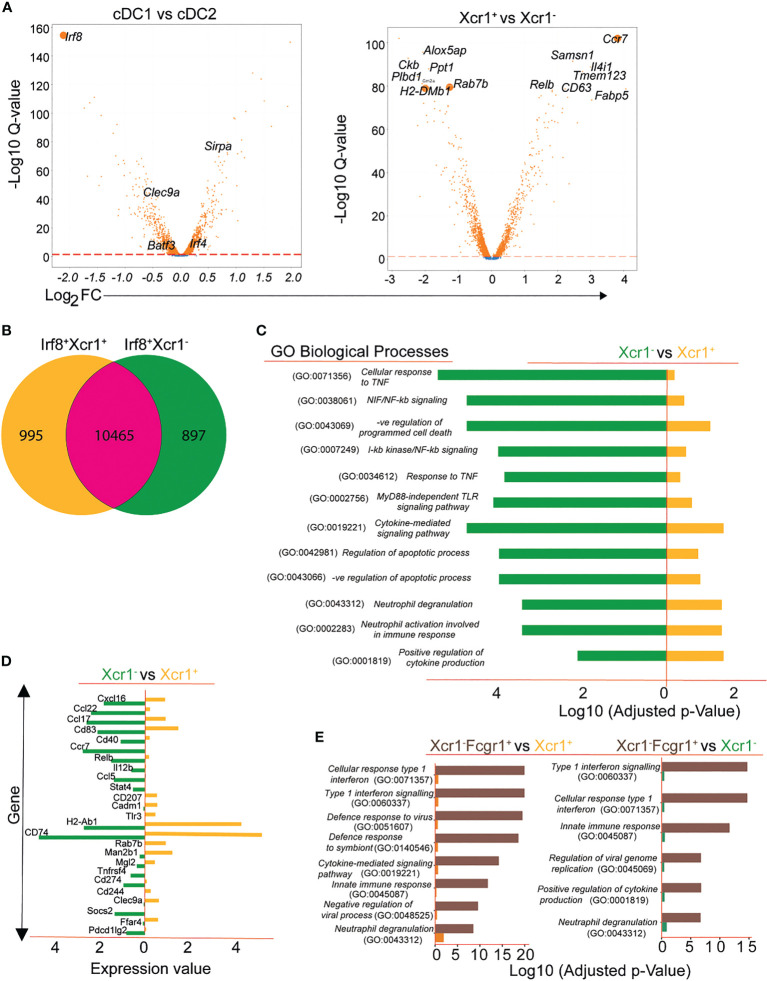
Transcriptional patterns and functional heterogeneity of cDC1 clusters. Conventional DC1 were characterized based on expression of Irf8 and Batf3 and DC2 based on Irf4 and SIRP-α **(A)**. Volcano plot showing subdivision of Irf8-expressing cDC1 based on Xcr1 expression (**A**, right). Differential gene expression depicted using Venn diagram showing DGEs between Xcr1+ and Xcr1- cDC1 **(B)** and gene ontology results of various biological processes differentially regulated in the 2 cDC1 clusters **(C)**. A selection of functionally important genes differentially expressed between Xcr1+ and Xcr1- cDC1 clusters **(D)** and a comparative analysis of biological processes between Xcr1^-^Fcgr1^+^ cDC2 cluster and the cDC1 clusters Xcr1^+^Irf8^+^ and Xcr1^-^Irf8^+^ **(E)**.

We first focused on data of the allergen-naïve control mice to characterize the identified clusters. We carried out differential gene expression analysis between cDC1 and cDC2 as depicted by the volcano plot (**Figure 2A** left). Further gene analysis shows the Xcr1^+^ and Xcr1^-^ clusters of cDC1 sub population also express different genes (**Figure 2A** right). In order to understand functional differences between Xcr1^+^Irf8^+^Batf3^+^ cDC1 and Xcr1^-^ Irf8^+^Batf3^+^ cDC1 clusters, we assessed differential gene expression in allergen-naïve control mice that had not been referred to the allergic asthma model or allergen tolerance model (**Figure 2B**; **Supplementary FigureS2**). We identified 1892 genes that were differentially expressed between the two cDC1 clusters with the Xcr1^+^ subset expressing 995 and the Xcr1^-^ subset expressing 897 unique genes (**Figure 2B**). In order to understand differences between the two clusters, we carried out enrichment analysis of gene sets of both clusters for biological processes on Gene Ontology (GO) terms and observed significantly different enrichment patterns for the processes between the two clusters (**Figure 2C**). Based on enrichment scores, we observed several GO Terms associated with inflammatory responses that were enriched in Xcr1^-^Irf8^+^Batf3^+^ cDC1 transcriptome as compared to the Xcr1^+^Irf8^+^Batf3^+^ cDC1 transcriptome (**Figure 2C**). This observation points towards a more activated state or propensity for activation of the Xcr1^-^ cluster prior to antigen exposure.

For efficient stimulation of resting T cells, DCs are equipped with a repertoire of costimulatory molecules (1, 2, 4, 41–43). Analysis of the transcriptomic patterns of Xcr1^+^ cDC1s and Xcr1^-^ cDC1s revealed high expression of markers associated with T cell activation (*MHCII* complex, *H2-Ab1*, *Cd74*) and *Clec9a* in the Xcr1^+^ cDC1 cluster and high expression of genes associated with inflammation such as *Relb*, *Ccl22*, *Ccl5* and *Socs2* amongst others in the Xcr1^-^ cluster (**Figure 2D**). Thus, transcriptional expression of the chemokine receptor Xcr1 identifies a cDC1 cluster with tolerogenic properties while absence of Xcr1 identifies a pro-inflammatory cluster of cDC1 as indicated by high expression of genes relevant for migration, antigen uptake and presentation as well as maximal T cell stimulation. As we have mentioned above, we observed a third cluster which expressed *Irf8* in the lungs of mice subjected to either asthma or tolerance protocol. The third *Irf8* expressing cluster in allergen-exposed mice shares many similarities with a recently described inflammatory cDC2 (39) as shown by high *Fcgr1* expression and genes of the interferon-signaling pathways (**Figures 1C**, **S2**). This was also evident when we carried out a comparative analysis of significantly regulated gene ontology terms of this cluster with both the Xcr1^+^ and Xcr1^-^ cDC1 clusters (**Figure 2E**).

### Elevated frequencies of pulmonary Xcr1^-^Irf8^+^Batf3^+^ cDC1 in experimental allergic asthma

**Figure 3:**
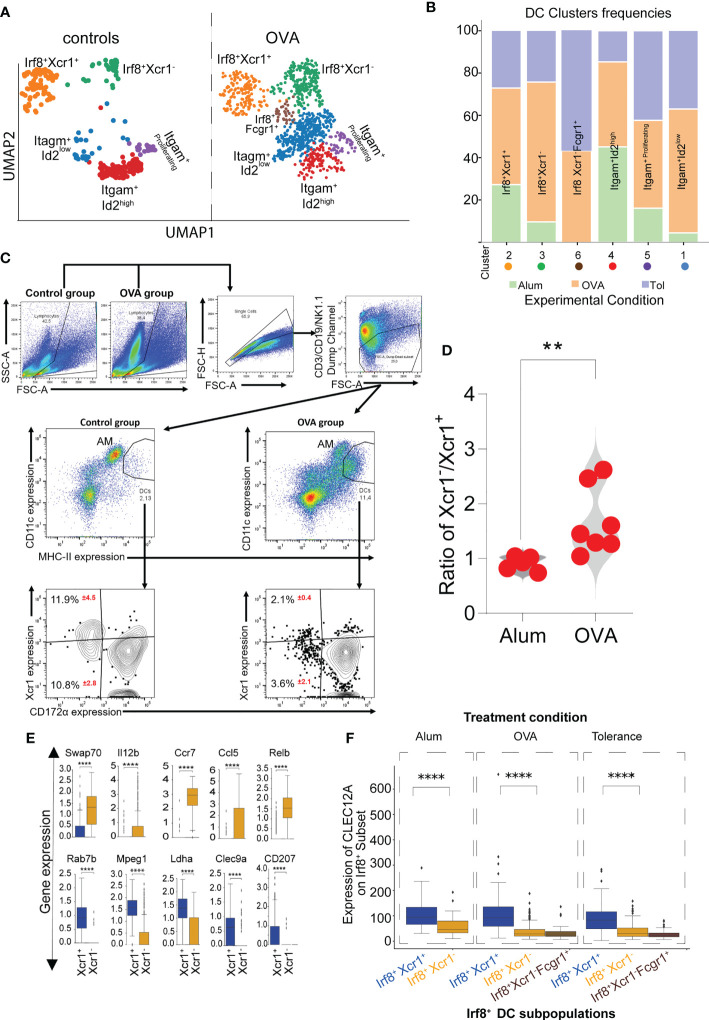
Impact of inflammation on Xcr1^+^ and Xcr1^-^ cDC1 clusters revealed by scRNASeq. UMAP showing cDC clusters as seen in mice subjected to either allergen-naïve control procedure or experimental asthma **(A)**. Frequencies of each DC cluster in the control, OVA-induced asthma or tolerance models **(B)**. Characterization and analysis of Xcr1-expressing cDC1 in mice subjected to either control or asthma protocol showing a shift in subset distribution **(C)** and violin graph analysis indicating that the changes in frequencies occur in Xcr1^-^ cluster in animals subjected to the experimental asthma **(D)**. Analysis of selected genes relevant for functional differences between the Xcr1^+^ and Xcr1^-^ cluster **(E)** and expression of CLEC12A on Irf8^+^ subpopulations of cDCs **(F)**. p value in **(D)** was calculated using the non-parametric Mann-Whitney Test and the dots indicate the number of animals in each group. AM (Alveolar macrophages), DCs (dendritic cells). **Represents a p value less than 0.01, **** Represents a p value less than 0.0001.

Whereas the Xcr1^+^ Irf8^+^Batf3^+^ cDC1 showed similar frequencies in all experimental conditions, Xcr1^-^Irf8^+^Batf3^+^ cDC1 frequencies were elevated in the allergic asthma model compared to the allergen tolerance model and controls (**Figures 3A, B**). Based on our data, we hypothesize that the Xcr1^-^ cluster of Irf8^+^Batf3^+^ cDC1s represents an inflammatory type 1 cDC cluster while the Xcr1^+^ cluster, whose frequencies remained similar in all three treatment conditions, represents classical cross-presenting cDC1 with a homeostatic function (**Figure 3B**). To further analyze the influx of pro-inflammatory Xcr1^-^ Irf8^+^Batf3^+^ cDC1s in the lungs of mice with an allergic asthma-like disease (**Figure 1A**), we compared frequencies of Xcr1^+^ and Xcr1^-^ cDC1s *via* flow cytometry in our experimental allergic asthma model and showed an increase in frequencies of pro-inflammatory Xcr1^-^ CD172α^-^ cDC1 in lungs of animals subjected to the asthma model (**Figures 3C, D**). Further transcriptome analysis of single cell data showed that the Xcr1^-^Irf8^+^Batf3^+^ cDC1 expressed significantly higher levels of *Swap70*, *Il12b*, *Ccr7*, *Ccl5* and *Relb* transcripts which confirms the pro-inflammatory properties of this cDC1 cluster (**Figure 3E**). In contrast, Xcr1^+^Irf8^+^Batf3^+^ cDC1s were characterized by high levels of genes associated with induction of tolerance such as Clec9a which was significantly reduced in the Xcr1^-^Irf8^+^Batf3^+^ cDC1 cluster (**Figure 3E**) and higher cell surface expression of Clec12A as assessed by oligonucleotide barcoded anti-Clec12A antibody (**Figure 3F**). In accordance with known literature, we also observed an influx of cDC2s in animals subjected to either experimental asthma or tolerance models (30, 40, 44) (**Figures 3A, B**). Furthermore, Xcr1^-^Irf8^+^Fcgr1^+^ cDC2 cluster appeared only in allergen-exposed animals and not in allergen-naïve control mice (**Figures 3A, B** **)** indicating that this cluster represents the inflammatory cDC2 as previously reported (39).

### RNA velocity and latent time analysis illustrates differences in temporal trajectories of Xcr1^+^ and Xcr1^-^ cDC1 clusters

**Figure 4:**
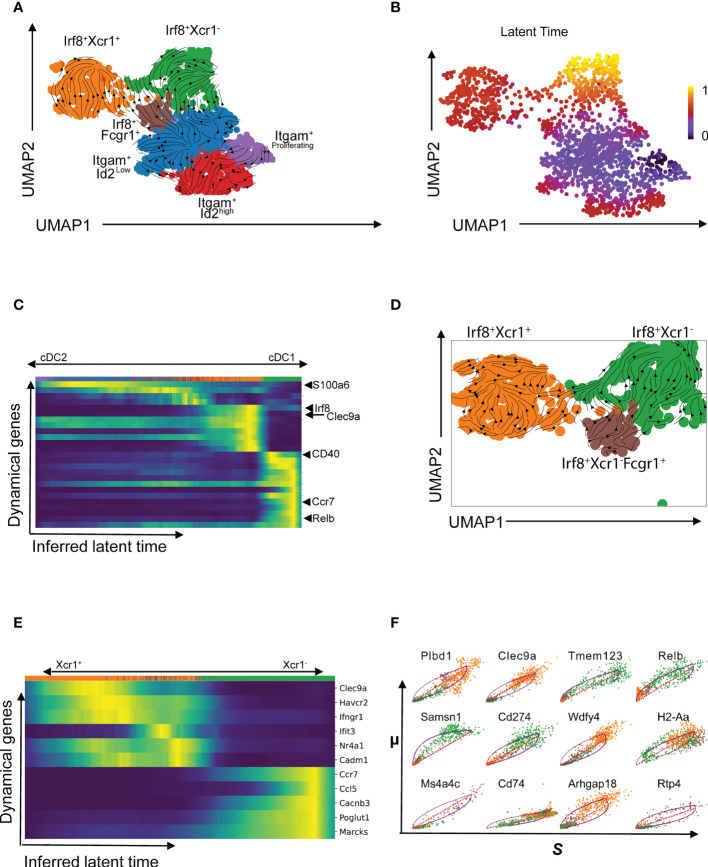
Temporal patterns of pulmonary cDCs using RNA velocity. RNA velocity was applied to understand temporal patterns of various cDC clusters identified using scRNASeq **(A)**. Latent time showing temporal patterns based on transcriptional activities in identified clusters **(B)** and heatmap of velocity-driving genes with annotations of a few at the positions where they appear for the different cDC clusters **(C)**. RNA velocity graph for the Irf8-expressing sub-clusters **(D)** and dynamic model showing putative driver genes for the Irf8-expressing subgroup with annotations of a selected gene set **(E)**. Comparative analysis of selected putative driver genes of the Xcr1^+^ (orange cluster) and Xcr1^-^ (green cluster) comparing spliced and unspliced mRNA ratios **(F)**.

**Figure 5:**
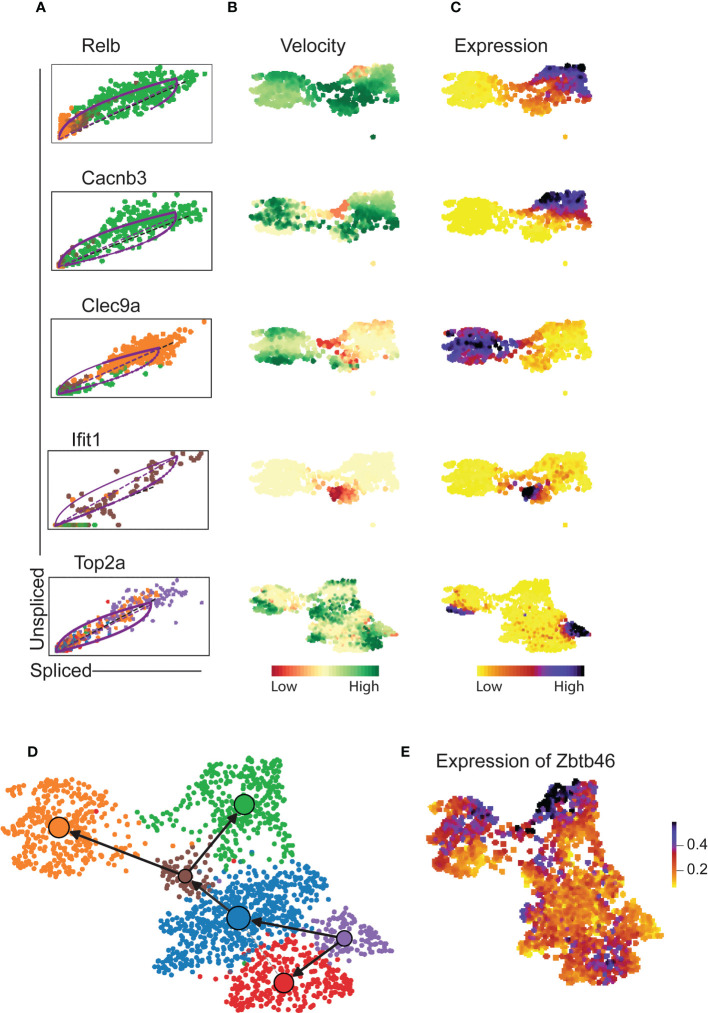
Cluster-specific differential velocity expression patterns of pulmonary cDCs. RNA velocity length and expression patterns of genes Relb, Cacnb3, Clec9a, Ifit1 and Top2a on pulmonary cDCS **(A-C)** showing differences in both velocity and expression patterns on various clusters of cDCs. Partition abstraction graph (PAGA) showing trajectories of the clusters embedded into the velocity graph **(D)** and expression of Zbtb46 on various clusters **(E)**. The solid black arrows represent transitions with high confidence based on the velocity data. Lack of arrows in **D** depicts lack of velocity connectivity between the clusters.

Velocity analysis is a powerful tool to investigate cell fate trajectories, for example during maturation. Gene specific profiles of unspliced and spliced mRNA transcripts are applied in analysis of RNA velocities to derive transcriptional dynamics and estimate future states of a cell (45). Here, we used velocity analysis derived from the single-cell RNAseq data to investigate the temporal relationship of Xcr1^-^Irf8^+^ cDC1s and Xcr1^+^Irf8^+^ cDC1s clusters. *Xcr1* downregulation as well as expression of migration associated markers such as *Ccr7* have been shown to indicate maturation and activation of cDC1 (26). These studies imply existence of a temporal relation between the Xcr1^+^ and Xcr1^-^ cDC1 clusters, we have identified in our study. To test this hypothesis, we applied the likelihood-based dynamical model to determine RNA velocities and combined this with the transcriptome-based clustering (**Figure 4A**). RNA velocity as well as latent time analysis of both cDC1 and cDC2 clusters show distinct patterns (**Figures 4A, B**). Importantly, dynamics-driving genes were unique between various clusters which shows that these clusters have individual trajectories (**Figures 4C, D**) as well as functional capabilities. The functional difference between Xcr1^+^ and Xcr1^-^ cDC1s can easily be observed through differential patterns of functionally important genes such as *Clec9a*, *Ccr7*, *Relb* and *Cd40* amongst others which also seem to define RNA velocity pattern of these two clusters (**Figures 4C, E**). Positive velocity of genes such as *Clec9a*, *Rab7b*, *Wdfy4*, *Ppt1*, *Cadm1*, *Havcr-2* (Tim-3) delineated cross-presenting and tolerogenic cDC1 as previously reported (46) whereas in contrast, genes associated with inflammation and activation such as *Relb*, *Ccr7*, *Ccl5*, *Cacnb3*, *Cd274* (PD-L1) were among the top driver genes for Xcr1^-^ Irf8^+^ cDC1 cluster (**Figure 4F**, **5A–C**). Cells of the Xcr1^-^Irf8^+^Fcgr1^+^ cDC2 cluster, which appeared only in allergen-treated animals of the asthma and tolerance model, showed high transcriptional activity in interferon-induced genes such as *Ifit1*, *Ifrd1*, *Map3k14* and *Infgr1* confirming the activated and pro-inflammatory character of this cell population (**Figures 5A–C**) as previously described (30, 39). To confirm uniqueness of the trajectories of the Xcr1^+^ and Xcr1^-^ clusters, we carried out partition-based graph abstraction (PAGA) analysis which confirmed lack of connectivity in trajectories of the two clusters as depicted through absence of continuous transition from Xcr1^+^Irf8^+^cDC1 to Xcr1^-^Irf8^+^cDC1 (**Figure 5D**). Interestingly, our trajectory analysis also showed a temporal connectivity between the Xcr1^-^Irf8^+^Fcgr1^+^ cDC2 and the two cDC1 clusters probably as a result of the acquisition of cDC1 transcriptomic features by this inflammatory cDC2 cluster (**Figure 5D**). The Xcr1^-^ Irf8^+^Fcgr1^+^cDC2 cluster was situated between Itgam^+^Id2^low^ cDC2s and the two cDC1 clusters, showing connectivity with both cell populations. Nevertheless, according to PAGA analysis, the connectivity between the Itgam^+^Id2^low^ cDC2s and Xcr1^-^ Irf8^+^Fcgr1^+^ cDC2 was of low confidence as depicted by the dotted line. In contrast, Xcr1^-^Irf8^+^Fcgr1^+^ cDC2 showed trajectories to the two cDC1 populations indicating the similarity towards the cDC1 clusters due to upregulation of cDC1 genes such as *Irf8* and downregulation of cDC2 genes such as *Irf4*, *Itgam* and *Sirpa* as depicted in **Figure 2A** above. Lastly, the Itgam^+^ cDC2 clusters showed a clear trajectory from a proliferating Itgam^+^ cluster towards both the Id2^high^ and Id2^low^ cDC2 clusters with high connectivity and high confidence (**Figure 5D**).

Overall, expression of the transcription factor *Zbtb46* confirmed the authenticity of all six clusters as cDCs (**Figure 5E**). Thus, the velocity-based trajectories support the hypothesis that Xcr1^+^Irf8^+^ cDC1 and Xcr1^-^Irf8^+^ cDC1 are two distinct cDC1 clusters with independent cell fate trajectories.

## Discussion

The current paradigm regarding cDCs supports the notion that there is a single population of cDC1 which depends on the transcription factors *Irf8* and *Batf3*. This population has surface expression of Xcr1 (12, 15) and can cross-present antigen. Importantly, this single population is capable of inducing either inflammatory or tolerogenic responses (16, 23, 27, 28), a duality of function that is currently attributed to the stimuli from the local environment and activation status (47, 48).

In this study, we investigated cDC subpopulations by single-cell RNA-sequencing and report evidence for the existence of two constitutively present *Irf8^+^* and *Batf3^+^* conventional cDC1 clusters in the murine lung with distinct functional properties regarding tolerance induction and inflammation. We identified the well-known Xcr1^+^Irf8^+^Barf3^+^ cDC1 cluster as well as an Xcr1^-^Irf8^+^Batf^+^ cDC1 cluster which was present in allergen-naïve control mice and which expanded at least in frequencies during type-2 inflammation induced in a murine allergy model. Furthermore, we identified the Xcr1^-^ cDC1 population by surface staining using flow cytometry and confirmed its enhancement during allergen-induced inflammation when compared to the Xcr1^+^ cDC1 cluster.

Consistent with its increase during inflammation, the Xcr1^-^Irf8^+^ cluster of cDC1 showed a transcriptomic pattern supporting enhanced migratory, antigen presentation and inflammatory capabilities as shown by elevated expression of *Ccr7*, *Cd74*, *MHC-II*, *Ccl5*, *Il12b* and *Relb*, factors that have previously been attributed to immunogenic DC (26). This is in contrast to the well-described Xcr1^+^Irf8^+^ cluster, which expresses high levels of *Clec9a*, *Cadm1*, *Btla* and *Pbx1* supporting their primary role in induction of tolerance and maintenance of homeostatic stability.

Xcr1 downregulation in cDC1 has been reported by other authors as well (26, 30, 49) which is not necessarily associated with loss of Xcr1 on the cell surface as shown by data combining single-cell transcriptomics with cellular indexing of transcriptome epitopes (CITE)-seq (49, 50). Using flow cytometry, we could additionally identify pulmonary cDC1 without Xcr1 surface expression in lungs of allergen-naïve control animals which expanded after allergen exposure confirming earlier observations of CD11b and Xcr1 double negative cDCs (51, 52). Moreover, downregulation of *Xcr1* expression in cDC1 has been shown to be associated with immunogenic or homeostatic maturation and activation (26, 49) or adoption of a regulatory phenotype (49). It is however still unclear if Xcr1-Irf8+Batf3+ cDC1 develop from Xcr1+Irf8+ cDC1 by maturation/activation or if they constitute an independent cell population (47). To explore these two options, we used velocity analysis of single-cell RNAseq data to examine the temporal dynamics of our DC clusters. In cDC2s we found a clear trajectory from proliferating cDC2 cells to other clusters expressing *Itgam* and *Sirpa*, however in cDC1s we did not find a directional trajectory from Xcr1+Irf8+ cDC1s to Xcr1-Irf8+ cDC1s which supports our hypothesis of a distinct Xcr1-Irf8+ cDC1 cluster rather than a maturation process.

In allergen-treated animals, we identified a cDC cluster which showed high resemblance to a recently described inflammatory cDC2 population found in murine lungs after viral infection or allergic type 2 inflammation (30). Similarity was established by expression of *Fcgr1*, upregulation of *Irf8* while retaining cDC2 gene expression as *Irf4*, *Itgam* and *Sirpa* and induction of genes of the interferon I pathway. Our velocity analysis supported the “hybrid” state of this newly described inflammatory cDC2 populations as trajectories were seen from Id2^low^ cDC2 population to the Xcr1^-^Irf8^+^Fcgr1^+^ cDC2 as well as from the Xcr1^-^Irf8^+^Fcgr1^+^ cDC2 to both cDC1 populations. Interestingly, PAGA analysis indicates that the connectivity between the two cDC2 populations was of lower confidence, than between the Xcr1^-^Irf8^+^Fcgr1^+^ cDC2 and the two cDC1 populations which supports the strong cDC1 features of this hybrid inflammatory cDC2 population (39).

We specifically analyzed the Irf8^+^Xcr1^-^Fcgr1^+^ cDC2 together with the cDC1s due to its expression of Irf8 and Batf3 and thereby, confirmed the uniqueness of this cluster alongside the *bona fide* cDC1 clusters we have described in this study. Thus, we see this work as an addition to recent results demonstrating that cDC1 and cDC2 populations clearly show more fluidity as previously perceived (30, 49).

Thus, in line with our data, it is plausible to speculate at least in the murine lung, existence of two phenotypically distinct cDC1 clusters which are present in steady state and are equipped to take on distinct roles regarding the two major tasks of cDC1, namely maintenance of tolerance and induction of an immunogenic response. Transcriptomic profiles confirm the role of Xcr1^+^Irf8^+^ cDC1s for induction of tolerance and maintenance of homeostasis, while the transcriptome profile of the Xcr1^-^Irf8^+^ cDC1 cluster supports an inflammatory function such as enhanced antigen processing, migration, co-stimulation and activation of T cells. Increased protein expression of Clec12A on Xcr1^+^Irf8^+^ cDC1 additionally supports a role in maintaining homeostasis and prevention of uncontrolled inflammation (36, 38). Importantly, velocity based temporal pattern analysis showed clearly that both Xcr1^+^Irf8^+^ and Xcr1^-^ Irf8^+^ cDC1s have different fate maps and thereby constitute distinct clusters with no evidence of plasticity amongst them.

Nevertheless we have to report some limitations of this study. Firstly, our single-cell data did not include data of untreated naïve animals; we only included the appropriate control group for the experimental set-up and secondly, overall, our data is mostly observational. Experiments to track time kinetics and phenotypical plasticity of the various cDC clusters as well as their functional role are still necessary to support our finding. Developmental kinetics could for example be analyzed using barcoded myeloid progenitor cells to track the evolution of cDC1 clusters under steady state and inflammation. Furthermore, confirmation of the functional diversity of the cDC1 clusters should be assessed by analyzing the cross-presenting and tolerogenic capacity of Xcr1+ cDC1s compared to the pro-inflammatory function of Xcr1- cDC1s. Still, despite these limitations we generated our data using a robust experimental model in an unsupervised manner to test a specific hypothesis regarding the Irf8-expressing cDCs. We think based on our experimental set up and analysis strategy, we have not only confirmed previous work indicating plasticity within various cDC clusters but also addressed the question of diversity within the cDC1 subpopulation. However, our work still leaves the question regarding influence of the cytokine milieu in determining functional dichotomy of cDC1 open. Future work should attempt to dissect the role of surrounding environment on various cDC1 clusters we have reported.

Despite the limitations, based on our data it is still plausible to conclude that while the Xcr1^+^ cDC1 cluster is equipped to constitutively induce tolerance especially due to the antigen cross-presentation-ability of cDC1 (3, 4, 17, 20, 23, 28, 53), the Xcr1^-^ cDC1 cluster is immunogenic and expands in murine lungs during inflammation analogous to the inflammatory cDC2. We argue that the Xcr1^-^ cDC1 cluster is not just an activated state of Xcr1^+^ cDC1s (26) but expands on demand for an optimal antigen presentation to T cells as shown through the repertoire of genes highly expressed on them. Hence, as much as cDCs tolerogenicity and immunogenicity may be programmable based on the surrounding *milieu*, our high-dimensional single cell analysis supports dichotomy of cDC1s into either tolerogenic or immunogenic clusters both in inflammatory and non-inflammatory conditions *in vivo*. Finally, we conclude, that efforts to use cDCs for immune-modulation consider this heterogeneity in order to appropriately direct antigen delivery to the right cell type for an effective generation of an immune response.

## Materials and methods

### Animals

The Committee on Animal Welfare in the State of Lower Saxony (LAVES) approved all animal protocols that were used in this study. Female age matched C57BL/6J mice obtained from Charles River Laboratories (Sulzfeld, Germany) used in this study were maintained in the animal facility at Hannover Medical School, Hannover, Germany.

### Induction of allergic asthma and allergen-tolerance

We have established an experimental asthma protocol in which mice are sensitized intraperitoneally (i.p.) with Polymyxin-treated Grade V OVA (20 µg) adsorbed to 2 mg of an aqueous solution of aluminium hydroxide and magnesium hydroxide (Alum; Fischer Scientific International) followed by repeated intranasal (i.n.) challenges (20 µg OVA in 40 µl normal saline). Control mice received Alum without OVA i.p. and 0,9% NaCl i.n instead of OVA (54). In order to induce tolerance, mice inhaled 500 µg of OVA on days 6 and 3 prior to starting the asthma protocol as depicted on **Figure 1A**.

### Flow cytometry based characterization of pulmonary antigen presenting cells

Lung DCs and alveolar macrophages (AM) were sorted as previously described (55). Briefly, single cells were isolated from lung tissue by digestion of the lungs using a mixture of collagenase and DNAse (Milteny). Cells were stained with CD11c, CD11b, MerTK, CD64, Ly6C and MHC-II. Pulmonary DC were isolated by Flourescence Activated Cell Sorting using a FACSAria (Becton-Dickinson). DCs were identified based on high expression of MHC-II and CD11c within MerTK, CD64 and Ly6c negative population previously described (55, 56). Flow cytometry analysis of DCs was done based on CD11c, MHC-II, Xcr1 and CD172α.

### Single cell mRNA sequencing

Prior to mixing, cells sorted from each condition were tagged using hashtag derived oligonucleotides (HTOs) from BioLegend, United States. The cell hashing based experimental approach was used to demultiplex pooled cell samples. Accordingly, three different cell types originating from independent cell suspensions were pre-incubated with three different TotalSeq™-A Hashtag Derived Oligo (HTO) antibodies as follows: control group with (barcode A0301; catalog #155801), Asthma group (barcode A0302; catalog #155803) and tolerance group (barcode A0303; catalog #155805). In addition, all cells were further stained with antibody-derived tag CLEC12A (barcode 0825, BioLegend, United States catalog #143407) according to the manufacturer’s manual. Equal numbers of pre-incubated cells were pooled and loaded on one 10x Genomics lane. One of such pooled cell sample generated applying the cell hashing approach is referred to as one ‘master-sample’ and in order to test technical robustness we prepared two ‘master-samples’ with each master sample representing cells sorted from three different treatment conditions control, asthma and tolerance as indicated in **Figure 1A**.

### Library generation

Library preparation for single cell mRNA-Seq analysis was performed according to the Chromium Single Cell 3′ Reagent Kit v3 User Guide (Manual Part Number CG000183 Rev A; 10x Genomics). Thus, 1.6-fold excess of cells was loaded onto the 10x controller in order to reach a target number of 9.000 cells per ‘master sample’. Fragment length distribution of generated libraries was monitored using ‘Bioanalyzer High Sensitivity DNA Assay’ (5067-4626; Agilent Technologies). Quantification of libraries was performed by use of the ‘Qubit^®^ dsDNA HS Assay Kit’ (Q32854; ThermoFisher Scientific).

### Sequencing run

Generated libraries were pooled accordingly, denatured with NaOH and finally diluted to 1.8pM according to the ‘Denature and Dilute Libraries Guide’ (Document # 15048776 v02; Illumina). 1.3 ml of denatured pool was sequenced on an Illumina NextSeq 550 sequencer using one High Output Flowcell for 75 cycles and 400 Million clusters (#20024906; Illumina). The flowcell capacity was utilized according to the molar proportions of individual libraries, adjusted as follows: Two mRNA expression libraries (‘master-samples’) with 40% of flowcell capacity each; two HTO libraries (‘master-samples’) with 5% capacity each; two ADT libraries (‘master-samples’) with 5% capacity each. Sequencing was performed according to the following settings: 28bp as sequence read 1; 56bp as sequence read 2; 8bp as index read 1; no index read 2.

### Raw data processing

The proprietary 10x Genomics CellRanger pipeline (v3.1.0) was used to perform the following steps: The BCL files were converted to FASTQ files with cellranger mkfastq using the respective sample sheet with utilized 10X barcodes of the ‘master-samples’. mkfastq wraps Illumina’s bcl2fastq and provides a number of convenient features in addition to the features of bcl2fastq. HTO and ADT data could be separated this way from expression data and end up in the ‘Undetermined’ fastq file fraction. Fastq data derived from the two replicates and 10x lanes (expression, HTO, and ADT) was separated based on individual barcodes, sequenced with index read 1. Based on the fastq files, the gene expression and feature barcoding data was processed using cellranger counts with default parameters. CellRanger was used to align read data to the reference genome provided by 10X Genomics (Mouse reference dataset 3.0.0; mm10) using the aligner STAR, counting aligned reads per gene, and calculating clustering and summary statistics for the ‘master-samples’. This step considers the feature barcoding to count HTO tags as well as ADT tags. The ‘master-samples’ were demultiplexed to get the ‘sub-samples’ by use of Seurat (v3.1.5) in R (v3.6.3) with a method based on the vignette “Demultiplexing with hashtag oligos (HTOs)” of Satjja Lab (https://satijalab.org/seurat/v3.1/hashing_vignette.html). Briefly, the hashtag data was log-normalized and clustered to end up in optimized separation of ‘sub-sample’ data based on hashtag signals, finally receiving individual lists of cell barcodes of the distinct ‘sub-samples’.

### Bioinformatic analysis

The analysis was done in Python (v3.7.8) using Scanpy (v1.5.1) (57). During the pre-processing of single cell data, the best practice guidelines by Luecken and Theis (2019) were followed (58), including the following steps: All cells with more than 20% of mitochondrial gene counts which have been shown to provide a hint of damaged cells, were excluded from downstream analysis. Cells with more than 20,000 counts or less than 12,000 genes were filtered out. Next, genes had to be expressed in at least five cells to be considered for further analysis. Scruplet (v0.2.1) (59) was applied to remove duplets. The data was normalized based on a deconvolution approach with Scran (60) and log-transformed. Combat (61), the Leiden-algorithm (57) and UMAP were utilised for the removal of batch effects, the cell clustering and cell cluster visualisation, respectively. Clusters were identified based on reported gene expression patterns and the expression of selected genes per cluster was evaluated to distinguish between cell types. For each cell cluster, cluster-specific genes were identified by a t-test with Benjamini-Hochberg correction decreasing the false discovery rate (57). After cell type identification, the dendritic cells were further specified by sub-clustering (Leiden, resolution = 0.5) and sub-cluster-specific genes were defined.

Differential gene expression between sub-clusters and gene set enrichment was done with the non-parametric Wilcoxon test (62) and EnrichR (63). RNA velocity analysis was done with scVelo’s (v0.2.1 (64) generalized dynamical model and. the matrices of spliced and unspliced counts were generated with Velocity (v0.17.17) (64). Sequencing data used in this paper can be found in the Gene Expression Omnibus (GEO) under accession no. GSE195899.

## Data availability statement

The datasets presented in this study can be found in online repositories. The names of the repository/repositories and accession number(s) can be found here: https://www.ncbi.nlm.nih.gov/geo, accession number: GSE195899.

## Ethics statement

The animal study was reviewed and approved by The Committee on Animal Welfare in the State of Lower Saxony (LAVES) approved all animal protocols that were used in this study.

## Author contributions

AJ, RG and GH conceived the project and designed the experiments. SG implemented the bioinformatics analysis together with DD, AJ, RG, SG, BL, SD, CH, OH, JM, AH, OB and DD conducted experiments or contributed to the analysis. GH secured funding for the project and provided supervision. AJ, RG and SG wrote the manuscript and designed the figures. All authors read and approved the manuscript.
