# Supplementary material for: Single cell RNA sequencing reveals distinct clusters of Irf8-expressing pulmonary conventional dendritic cells
Source: Front Immunol. 2023 May 12;14:1127485. doi: 10.3389/fimmu.2023.1127485 (PMC10213693; doi:10.3389/fimmu.2023.1127485)
Supplement: Supplementary file 1 [file Image_1.pdf]

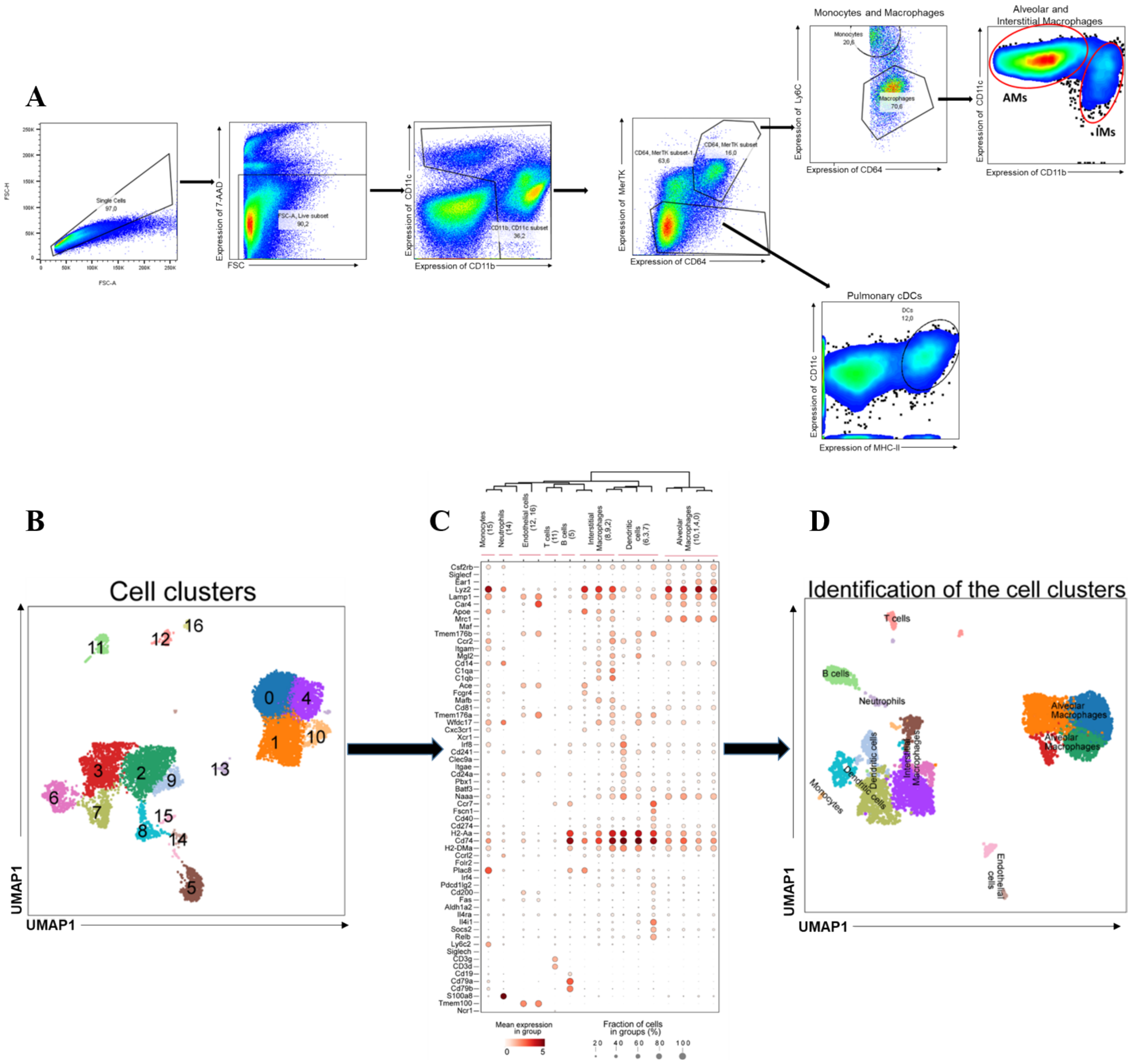

S1. scRNASeq reveals heterogeneity of murine pulmonary cDC. Gating strategy applied in sorting for pulmonary dendritic cells and macrophages **(A)** and unsupervised clustering showing various populations **(B)** which were identified (annotated) based on their transcriptomic patterns **(C, D)**.
