# Supplementary material for: Single cell RNA sequencing reveals distinct clusters of Irf8-expressing pulmonary conventional dendritic cells
Source: Front Immunol. 2023 May 12;14:1127485. doi: 10.3389/fimmu.2023.1127485 (PMC10213693; doi:10.3389/fimmu.2023.1127485)
Supplement: Supplementary file 2 [file Image_2.pdf]

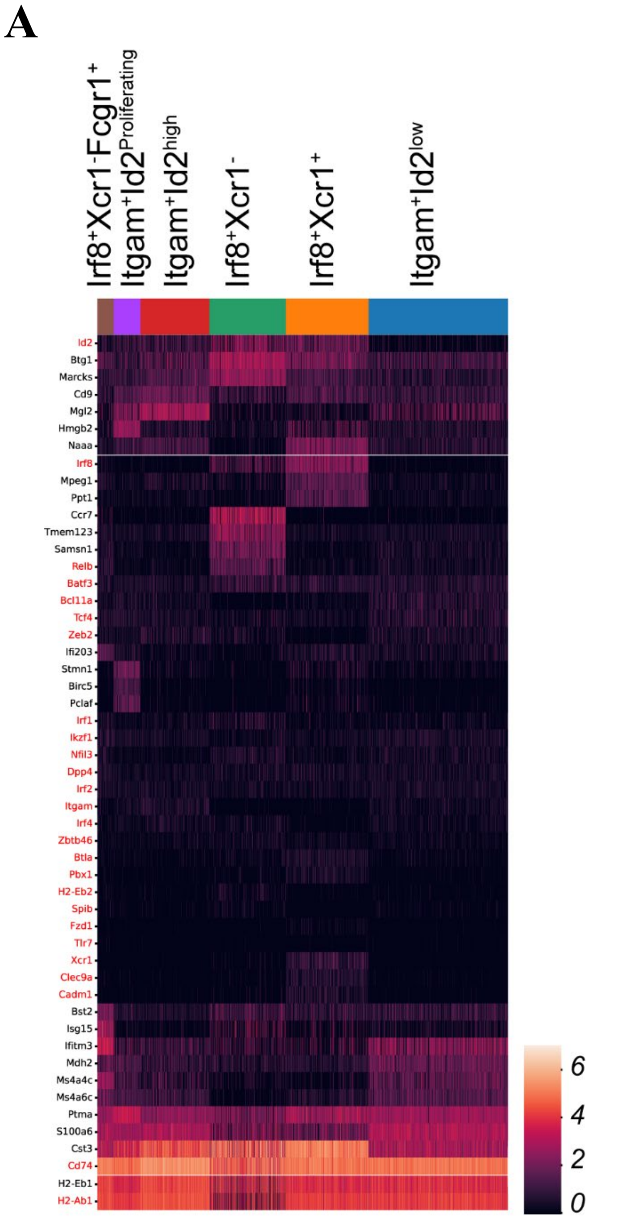

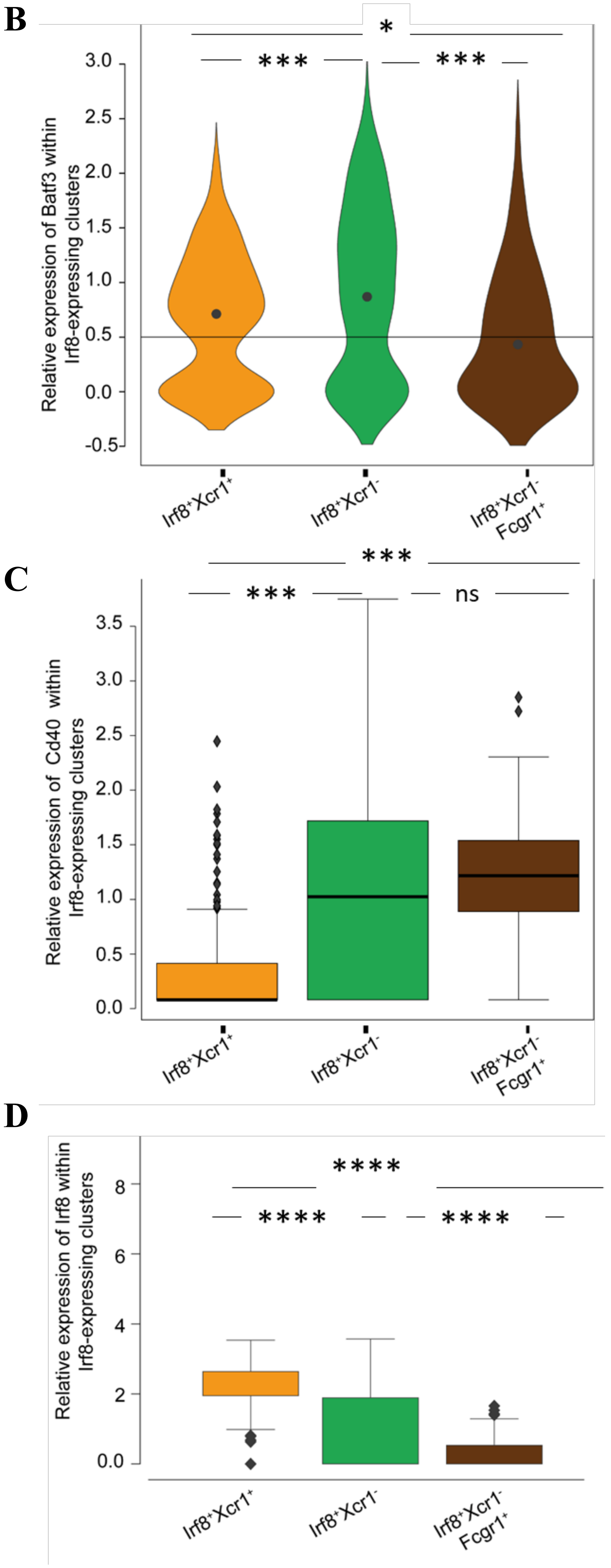

S2. Transcriptional patterns depicting heterogeneity of murine pulmonary cDC subpopulations. Heatmap showing expression of various cluster defining as well as functionally important genes expressed by cDC clusters **(A)**. Comparative analysis of Batf3 **(B)** Cd40 **(C)** and Irf8 **(D)** genes in Irf-8 expressing clusters.
